# Ethical Artificial Intelligence in Chemical Research and Development: A Dual Advantage for Sustainability

**DOI:** 10.1007/s11948-021-00325-6

**Published:** 2021-07-06

**Authors:** Erik Hermann, Gunter Hermann, Jean-Christophe Tremblay

**Affiliations:** 1 IHP – Leibniz-Institut für innovative Mikroelektronik, Frankfurt (Oder), Germany; 2 QoD Technologies GmbH, Berlin, Germany; 3 Université de Lorraine, Metz, France

**Keywords:** Artificial intelligence, Ethics, Research and development, Sustainability

## Abstract

Artificial intelligence can be a game changer to address the global challenge of humanity-threatening climate change by fostering sustainable development. Since chemical research and development lay the foundation for innovative products and solutions, this study presents a novel chemical research and development process backed with artificial intelligence and guiding ethical principles to account for both process- and outcome-related sustainability. Particularly in ethically salient contexts, ethical principles have to accompany research and development powered by artificial intelligence to promote social and environmental good and sustainability (*beneficence*) while preventing any harm (*non-maleficence*) for all stakeholders (i.e., companies, individuals, society at large) affected.

## Introduction

Artificial intelligence (AI) is a transformative power (re-)shaping businesses by providing new solutions to complex problems, increasing consistency and reliability, while decreasing costs and risks (Taddeo & Floridi, 2018). The “general purpose technology” character of AI can lay the foundation for innovations and capabilities (Brynjolfsson & Mitchell, 2017). Thus, AI has the potential to establish and bolster sustainable business models (e.g., Di Vaio et al., 2020; Mabkhot et al., 2021) and address major societal issues, among them, sustainable development as a focal challenge and objective of our time (Nishant et al., 2020; Vinuesa et al., 2020). AI approaches such as machine learning (ML) and deep learning facilitate the processing and analysis of massive amounts of structured and unstructured data (Jordan & Mitchell, 2015), which particularly benefits data-intensive research and applications. As chemical research has generated its insights from data from the very beginning (Gasteiger, 2020), AI is increasingly applied in various fields of chemical research including (synthetic) organic chemistry (e.g., de Almeida et al., 2019; Wei et al., 2016), toxicity prediction (e.g., Idakwo et al., 2019; Vo et al., 2020), quantum chemistry (e.g., Dral, 2020), (nano-)material science (e.g., Muratov et al., 2020), molecular design (e.g., Button et al., 2019), and drug discovery and design (e.g., Jiménez-Luna et al., 2020; Schneider, 2018, 2019; Zhang et al., 2017). AI in chemical research and development (R&D) can foster environmental and social good and embrace sustainability on two counts, that is, by developing more sustainable and ecofriendly substances and products on the one hand and by incorporating resource-efficient and sustainability-oriented methods in its R&D processes on the other hand (e.g., Ruiz-Mercado et al., 2012; van Wynsberghe, 2021).

Given the substantial impact of AI on the individual, economic, and societal level, AI development and use are accompanied by intensive discussions of guiding ethical principles by public and private institutions (Cowls et al., 2021; Floridi et al., 2018, 2020; Hagendorff, 2020; Jobin et al., 2019; Mittelstadt, 2019; Mittelstadt et al., 2016; Morley et al., 2020). However, the landscape of principles remains fragmented (Jobin et al., 2019) and translation into practice is needed (Mittelstadt, 2019; Morley et al., 2020). That is, recurring and prominent principles such as *transparency*, *beneficence*, and *non-maleficence* (Jobin et al., 2019) are normative, deontological, and high-order in nature (Hagendorff, 2020; Mittelstadt, 2019). However, translation into business and research practice might require trade-offs, context-dependent application, and consideration of different stakeholder interests. Stakeholders range from companies and organizations that (differently) interpret and apply ethical principles when developing and utilizing AI (Ryan et al., 2021), individuals that are directly or indirectly affected by AI, and eventually society at large (which is also impacted by environmental well-being). Accounting for the different stakeholders becomes particularly important when AI is operating between the priorities of promoting social good (i.e., *beneficence*) and preventing any harm (i.e., *non-maleficence*). Solving this tension to achieve a “dual advantage” for society (Floridi et al., 2018, p. 694) is at the core of the AI-for-social-good perspective (e.g., Cowls et al., 2021; Floridi et al., 2018, 2020; Taddeo & Floridi, 2018).

This conceptual study shows how accompanying ethical principles for the deployment of AI in chemical R&D can foster social and environmental good. Therefore, we present a chemical R&D process spanning cutting-edge chemical research that can substantially contribute to innovative products, methodological advances of AI, and guiding ethical principles. We rely our conceptual analysis on synthetic chemicals (i.e., pesticides) as illustrative R&D objects, since they are beneficent and maleficent at the same time and can be thus considered ethically controversial products. Thereby, our study contributes to the AI ethics literature by showing how ethical principles related to AI can be translated into business and research practice to promote environmental and social good while accounting for multiple stakeholder interests.

The remainder of our study is structured as follows. After briefly shedding light on how AI has evolved and is now applied in chemical R&D, we illustrate how chemical R&D powered by AI can be utilized for good through guiding ethical principles and consideration of all stakeholders affected. We conclude with a future outlook and a call for more collaborative, open science approaches to meet the global challenge of sustainable development.

## AI in Chemical R&D

Since (laboratory) chemical research has always accumulated enormous amounts of (experimental) data on chemical and physical properties, chemical reactions and structures, and biological activities, methods from computer science have been developed and utilized in chemistry starting in the 1960s (Gasteiger, 2020). They have been subsumed under the term artificial intelligence already then (Gasteiger, 2020). Quantitative structure property/activity relationship (QSPR and QSAR) modeling are long- and well-established computational approaches for analyzing chemical data (Gasteiger, 2020; Muratov et al., 2020). QSAR models have been historically applied to computer-aided drug discovery and are used to predict or design novel chemicals with desired properties by establishing linear or non-linear relationships between values of chemical descriptors computed from molecular structure and experimentally measured properties or bioactivities of those molecules (Muratov et al., 2020). Chemical discovery does not only pertain to finding a specific molecule, but also to identifying reaction pathways, interactions between molecules, optimizing catalytic conditions, eliminating adverse side effects, and various other factors. All of them require a statistical view on chemical substance design and discovery and thus give rise to ML techniques (Tkatchenko, 2020). For instance, hybrid methods uniting ML and rule-/expert-knowledge-based approaches and more advanced deep learning models have been developed for the molecular design of synthetic chemical entities with drug-like properties and for drug discovery, respectively (e.g., Button et al., 2019; Jiménez-Luna et al., 2020). With the availability of big data, drug discovery approaches increasingly move from ML to deep learning methods due to their computational power and capacity to handle massive amounts of data (Schneider, 2018; Zhang et al., 2017). Besides, AI approaches are gaining importance in predicting toxicity of drugs and chemicals (i.e., in silico toxicity prediction), because in vitro/*vivo* methods are often constrained by ethical considerations, time, budget, and other resources (e.g., Idakwo et al., 2019; Vo et al., 2020). Relatedly, life-cycle impacts of chemicals have been also shown to be assessable by means of AI (e.g., Song et al., 2017). Both toxicity of chemicals and substances and their life-cycle impact can be crucial factors that affect individual and environmental well-being. In the following, we illustrate how AI in chemical R&D can be harnessed to address these and other factors by accounting for ethical principles and the various stakeholders affected.

## Leveraging AI in Chemical R&D for Environmental and Social Good

**Fig. 1:**
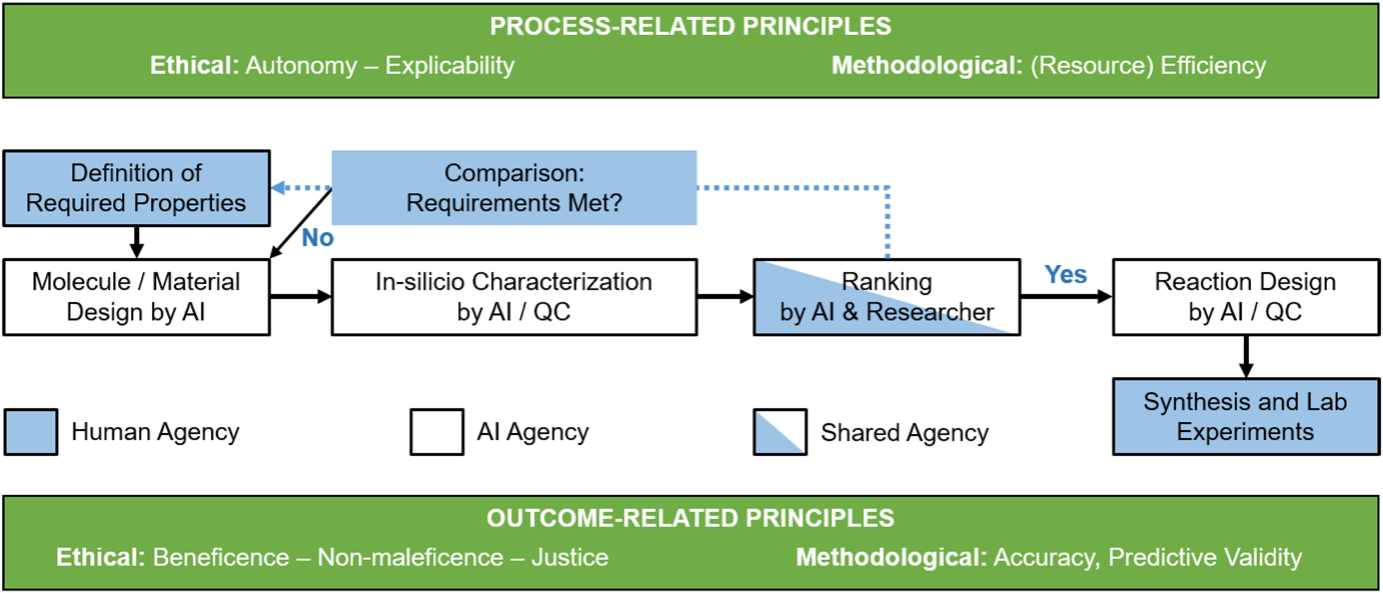
AI-driven R&D process. *QC* Quantum-chemical simulations

We account for the calls for nexus approaches and interdisciplinary research on sustainable development and climate change (e.g., Fuso Nerini et al., 2019; Schneider et al., 2019; Seele, 2016) by presenting an AI-driven chemical R&D process (see Fig. 1) and guiding ethical and methodological principles that relate to the R&D process and its outcomes (Burget et al., 2017). We argue that orientation on and observance of these guiding principles can contribute to both process- and outcome-related sustainability.

The proposed R&D process comprises the definition of the required properties, the AI-based molecular design and *in-silico* characterization of the relevant properties, the ranking of the most promising candidates, and respective AI-based reaction designs as basis for laboratory experiments. Synthetic chemicals (i.e., pesticides) are used as illustrative R&D objects for two reasons. First, they are central to cost-effective production of food and efficiency gains in agricultural systems (e.g., Pretty, 2018). Second, they simultaneously pose substantial environmental threats (Bernhardt et al., 2017). In other words, R&D of pesticides can be considered an ethically salient R&D context that requires ethically responsible conduct and anticipation of potential negative side-effects along the entire R&D process. Moreover, the deployment of AI applications in the sustainability context should account for all stakeholders potentially affected, particularly, given potential tensions of collective versus individual benefits and costs (Vinuesa et al., 2020). This multiperspectivity further accounts for the AI-for-social-good perspective (e.g., Cowls et al., 2021; Floridi et al., 2018, 2020; Taddeo & Floridi, 2018). Correspondingly, we focus on the AI ethics typology suggested by this stream of research (Floridi et al., 2018; Morley et al., 2020), that is, *beneficence*, *non-maleficence*, *autonomy*, *justice*, and *explicability*.

## Beneficence and Non-Maleficence

Scientists and experts state and warn that the imminent climate crisis is accelerating faster than expected and threatening natural ecosystems and humanity more severely than anticipated (IPCC, 2018, 2019; Ripple et al., 2020). Climate change presumably constitutes the most threatening global challenge for humanity (Coeckelbergh, 2021). That necessitates substantial increases of scale in endeavors to avoid untold suffering (IPCC, 2018; Ripple et al., 2020) and “bold solutions…that integrate environmental and societal objectives” (Arneth et al., 2021, p. 30882). Sustainability and sustainable development are pivotal to addressing these fundamental challenges. Both sustainability and sustainable development are widely used but polysemous concepts (Ben-Eli, 2018; Brown et al., 1987; Hopwood et al., 2005). Since mapping the different definitions and context-dependent meaning is out of scope of this paper, we simplistically refer to sustainability as a dynamic balancing between of human activity and environmental capacity by particularly limiting adverse environmental impacts and utilization of (natural) resources.

While some scholars argue that environmental sustainability is only worth pursuing for ethical reasons (Zagonari, 2020), others consider sustainability as an epistemic-moral hybrid (Schneider et al., 2019). Sustainability also constitutes an ethical principle and objective related to the development and deployment of AI (Jobin et al., 2019). According to the AI-for-social-good-perspective, sustainability is at the core of the *beneficence* principle, which inheres that AI should promote individual, social, and environmental well-being (Floridi et al., 2018). The *beneficence* principle is narrowly related—although not equivalent—to the tenet of *non-maleficence*. *Non-maleficence* incorporates the importance of safety, security, and privacy as well as the prevention of risks and any harm both accidentally/unintentionally (overuse) and deliberately (misuse) caused. Thus, it cautions against all potentially negative aspects and consequences of AI development and use (Floridi et al., 2018; Jobin et al., 2019).

AI in chemical R&D contributes to sustainability in two ways. First, AI simulations supplemented by quantum chemical (QC) predictions dematerialize and digitalize conventional lab experiments. That is, research questions (e.g., focusing toxicity and acidity) are addressed and answered by AI simulations (i.e., *in-silicio* characterization) instead of resource-intensive laboratory experiments. That can eventually result in substantial resource-efficiency gains and lower costs (e.g., due to minimization of material usage), that is, R&D process-related sustainability. The R&D process for synthetic chemicals such as pesticides become more resource-efficient and sustainable, because intended reductions in doses and environmental half-lives of active substances are usually accompanied by more complex molecular structures of synthetic chemicals. They in turn increase the resource utilization during the R&D process (Geisler et al., 2005). Of course, AI applications and systems have an ecological footprint themselves and can have rebound effects caused by energy consumption and emissions of AI development, production, and deployment (e.g., Dhar, 2020). That is, certain short-term trade-offs can occur, which is not unusual for sustainability efforts in general (e.g., De Neve & Sachs, 2020). Nevertheless, if AI-enabled simulations are going to be used at scale for a variety of research questions, we assume that the marginal (environmental) costs and impact of use will substantially decline and not outweigh their resource-efficiency gains in the long run.

Second, AI-based simulations relax the constraints of conventional laboratory research. Researchers can significantly extend the scope of research questions due to the computational power of AI and a vast array of scientific research and secondary, although often unstructured data. That is, simulations allow to simultaneously investigate a greater amount and diversity of relevant research questions and to substitute widespread, but inefficient one-parameter-at-a-time methods (e.g., Schneider, 2018). That is particularly relevant in the sustainability context when it comes to both effectivity (i.e., *beneficence*) and potential negative side effects (i.e., *non-maleficence*) of substances and prospect product candidates. Generally, the sheer volume, diversity, and intensity of use of chemicals can impede risk assessments and pose substantial environmental challenges (Johnson et al., 2020). Moreover, identifying and tracking chemicals and related (bioactive) transformation products in the environment and at ever-lower concentrations in human bodies is hampered by the complex mixture of thousands of chemicals the environment and humans are exposed to from multiple sources through multiple pathways (Escher et al., 2020). Therefore, anticipating and quantifying chemicals’ (detrimental) environmental impact requires comprehensive research activities, with synthetic chemicals like pesticides being no exceptions.

In light of calls for sustainable and ecological intensification of agricultural systems, that is, increased agricultural yields without the conversion of additional non-agricultural land and adverse environmental impact (e.g., Cassman & Grassini, 2020; Geertsema et al., 2016; Godfray & Garnett, 2014; Loos et al., 2014; Pretty, 2018; Pretty & Bharucha, 2014), synthetic chemicals like pesticides are a double-edged sword. Their beneficial role for pest management, crop yield, and food security (Cooper & Dobson, 2017) is compromised by pesticide resistance (e.g., Gould et al., 2018), reduction of biodiversity (e.g., Beketov et al., 2013; Dudley et al., 2017), and other negative externalities for human health and natural systems (Bernhardt et al., 2017; Pretty & Bharucha, 2015; Tilman et al., 2002). Conventional laboratory, experimental research does not sufficiently predict the individual and collective impact of synthetic chemicals on ecosystems, since their toxicity depend on reactions or interactions with other chemicals in natural environments, transformations by organisms, or exposure to natural light (Bernhardt et al., 2017).

**Fig. 2:**
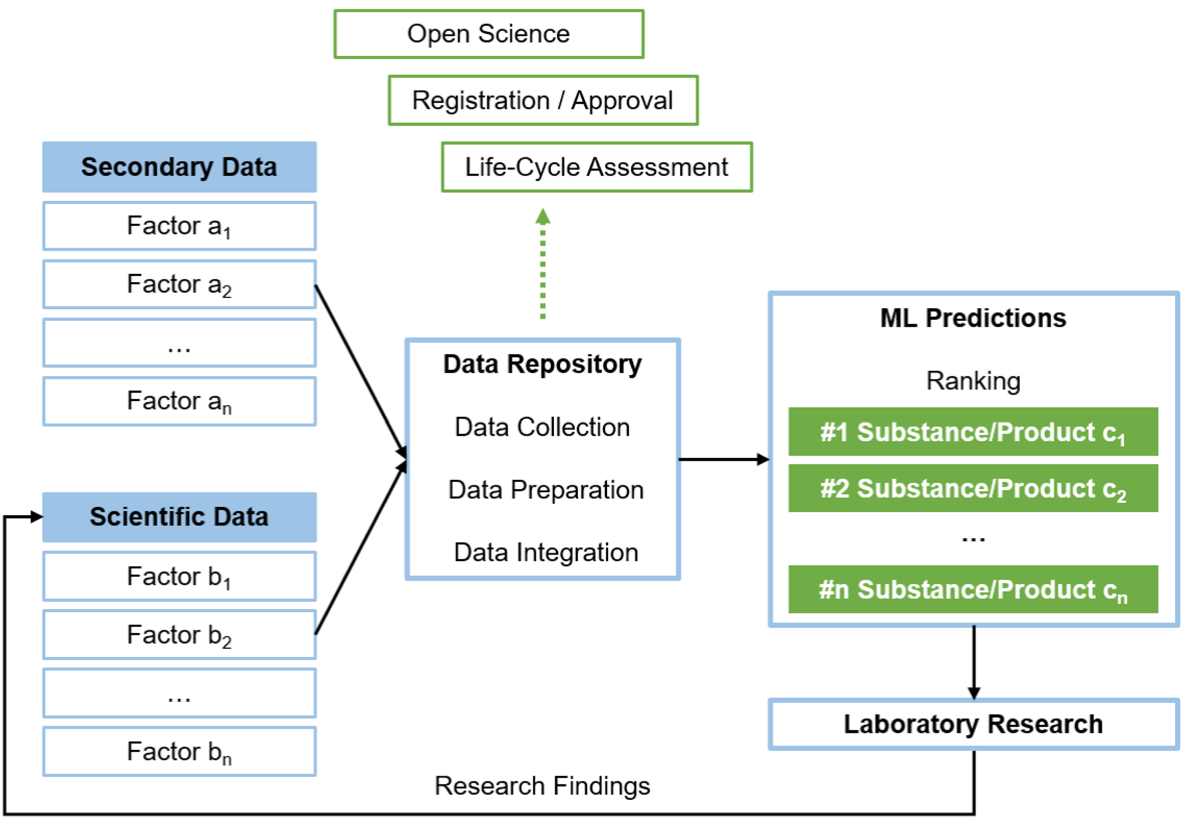
Data life cycle

AI with its self-learning capabilities and in combination with large-scale, increasingly rich, and high-dimensional research data (Vermeulen et al., 2020; Vinuesa et al., 2020) has the potential to account for these complex environmental interactions, interdependencies, and externalities. By feeding ML algorithms with relevant multifaceted scientific and secondary data (see Fig. 2), AI simulations in association with QC predictions will, in future, be able to define substance properties that are best-suited for areas of applications and surrounding circumstances and environmental factors (i.e., *in-silico* characterization and ranking) to maximize *beneficence* while limiting *maleficence*. Thereby, AI in chemical R&D also account for the green chemistry principles that include less hazardous chemical syntheses, designing safer chemicals, and inherently safer chemistry for accident prevention, among other things (e.g., Anastas & Warner, 1998; Anastas & Zimmerman, 2003; Erythropel et al., 2018; Zimmerman et al., 2020). Potential factors taken into consideration in ML models relate to life-cycle and environmental impact assessment categories and can comprise human toxicity, aquatic and terrestrial ecotoxicity, and acidification (Geisler et al., 2005).

However, a prerequisite for accurate and valid ML model predictions are curated consistent data sets (e.g., de Almeida et al., 2019; Schneider, 2018), since biases, inaccuracies, errors, and mistakes inherent in data could lead to biased results and false conclusions (Barredo Arrieta et al., 2020; Morley et al., 2020). Research findings of simulations, ML predictions, and subsequent laboratory research have to continuously complement research data bases, which, in turn, informs follow-up or related R&D. A data life cycle that enhances self-learning capabilities and fast feedback loops emerges. Furthermore, final and preliminary results have to be documented for subsequent life-cycle assessments, registration and approval processes, and to potentially provide them to other researchers or make them entirely publicly available by pursuing open science approaches (Rüegg et al., 2014). To optimize the re-use of research findings and scientific data, corresponding scientific data management structures should follow the FAIR principles for scientific data management, that is, Findable, Accessible, Interoperable, Reusable (Wilkinson et al., 2016). In this way, such a data life cycle (see Fig. 2) has intra-organizational epistemic and methodological advantages such as increased accuracy and validity of model predictions through broader data bases, feedback loops, and more comprehensive model training, but also provides benefits for external stakeholders. Accuracy and validity of ML predictions as well as ensuring *beneficence* and *non-maleficence* across stakeholders narrowly relate to the *justice* principle.

## Justice

By integrating broad and diverse data resources and a multi-stakeholder perspective, AI-based R&D processes incorporate the *justice* principle, which should also guide sustainable intensification (Loos et al., 2014) and environmental sustainability (Zagonari, 2020). The *justice* principles espouses fairness and the prevention of unwanted/unfair biases and discrimination, also amending past inequities (Jobin et al., 2019; Morley et al., 2020). *Justice* further entails sharing benefits and prosperity and fostering solidarity (Floridi et al., 2018), the latter being stipulated to be considered as a focal ethical principle on its own (Luengo-Oroz, 2019). In the AI-driven R&D context, research outcomes related to products but also scientific data have to be equally beneficial and non-discriminatory in respect to all stakeholders affected, within and across countries and regions. That is particularly important given the disproportionally adverse effects of climate change for poorer countries (IPCC, 2018) and the differences in pesticide use, efficiency of agricultural systems, and food security across countries (Cassman & Grassini, 2020; Pretty, 2018; Pretty & Bharucha, 2015). Developing countries are at the higher risks than developed countries, since there is no equity in the global distribution of chemical pollutants and their negative environmental externalities (Escher et al., 2020).

Bolstering and broadening R&D capacities through ML and QC simulations can be an initial step and future cornerstone of the mass customization of products deployed in agricultural systems. Thereby, agricultural systems’ idiosyncrasies can be taken into account in a fair and a cost- and resource-efficient way. Evidence and views of the benefits and costs of ethically controversial substances and products such as pesticides are far from unequivocal (Pretty & Bharucha, 2015). Hence, context-dependent evaluations considering all stakeholders and external (environmental) conditions are imperative and should inform judgments about interferences with ethical principles. In some circumstances, such assessments will require trade-offs between benefits (e.g., increase of crop yield enabling food security due to pesticide efficiency) and costs (e.g., adverse effects on biodiversity in certain environments). However, as human judgments can be error-prone, biased, and discriminating, so can AI predictions and inferences (Rich & Gureckis, 2019). As mentioned above, focal sources of biased predictions are biases in and skewness of underlying data (Barredo Arrieta et al., 2020; Morley et al., 2020; Vinuesa et al., 2020). Sources of biases include but are not limited to misleading proxy features (Barredo Arrieta et al., 2020) or sparse (small) data (de Almeida et al., 2019; Rich & Gureckis, 2019). Biases can further result from researchers themselves through personal preferences and biases and (chemical) education, which can also unwillingly narrow search spaces (de Almeida et al., 2019; Schneider, 2018).

## Autonomy

In light of human- and AI-induced biases, the *autonomy* principle and balancing human and AI agency becomes and will remain decisive in ethically salient R&D contexts. In the AI ethics context, *autonomy* features a meta-autonomy or a decide-to-delegate model, that is, “humans should always retain the power to decide which decisions to take” on their own or when to cede decision-making control (Floridi et al., 2018, p. 698). *Autonomy* in relation to AI applications and systems requires human agency (i.e., autonomous human decisions) and human oversight (Morley et al., 2020). As human researchers encounter difficulties to unambiguously grasp and determine utilities of R&D outcomes for different stakeholders, so does AI to an even higher degree (Butkus, 2020). Ethically controversial questions about environmental compatibility or toxicity of substances related to both humans and the environment necessitate human oversight and foresight (e.g., Floridi & Strait, 2020). However, human agency and decision-making do not only pertain to judging final AI predictions, but to the entire R&D process ranging from research question formulation, definition and assessment of required properties (dependent on areas of application), and evaluations of AI-based solutions. Correspondingly, de Almeida et al. (2019) noted that:

the right research questions must be asked prior to deploying the AI and its domain of applicability, advantages and limitations need to be well understood in order to assess the utility and appropriateness of a given algorithm for a particular task (p. 601).

Since artificial moral agency is still in its infancy (Cervantes et al., 2020), we propose a combination of human, AI, and shared agency along the R&D process (see Fig. 1). AI-driven chemical R&D can then incorporate multi-objective maximum-expected-utility concepts that are aligned to human values and ethical principles (e.g., Vamplew et al., 2018). Eventually, humans are in charge of equipping AI systems and their utility functions with ethical judgments capacities by decide about the respective AI design approach. Correspondingly, developers have to decide whether AI systems base their ethical decision-making on pre-defined ethical theories (top-down), on more flexible self-learning mechanisms based on certain values (bottom-up), or on both (hybrid) (Bonnemains et al., 2018; Cervantes et al., 2020).

To take on this challenge, collaboration and exchange with others stakeholders (e.g., Flipse et al., 2013) or ethicists, that is, an embedded ethics approach (e.g., Bonnemains et al., 2018; Brey, 2000; McLennan et al., 2020; Moor, 2005), to translate ethical principles into AI-powered business and research practice have to be contemplated. In the future, entirely autonomous AI decision-making in chemical R&D in the form of self-driving laboratories and closed-loop approaches (e.g., Häse et al., 2019; Muratov et al., 2020) are promising, but complicated in the case of ethically controversial R&D outcomes given the long way to go to achieve artificial moral agency (e.g., Cervantes et al., 2020).

## Explicability

Although researchers define required properties and should be involved in the research process (human agency), an understanding of how the AI works and predictions are derived is essential. That is at the core of *intelligibility*, the epistemological dimension of *explicability* (Floridi et al., 2018). The concepts *intelligibility*, *comprehensibility*, *interpretability*, *explainability*, and *transparency* are often used interchangeably and inconsistently (Barredo Arrieta et al., 2020), and are partly misconceived (Rudin, 2019). In a comprehensive review, Barredo Arrieta et al. (2020) identified *intelligibility*, that is, human understanding of a model’s function without any need for explaining its internal structure or underlying data processing algorithm, as the most appropriate conceptualization.

In the R&D context, the *intelligibility* principle is multi-faceted. While researchers that are directly involved in the AI development process and oversee the R&D process should have an in-depth understanding of underlying data and AI models’ structures and functions, a basic understanding might suffice for other internal organizational stakeholders. Otherwise, too complex and incomprehensible explanations and overly complicated decision pathways can impend (Ananny & Crawford, 2018; Rudin, 2019). From an external perspective, the *intelligibility* principle is regularly limited or diluted by proprietary boundaries and intellectual property right restrictions in case of commercial product development (e.g., Ananny & Crawford, 2018; Mittelstadt et al., 2016). However, provision of certain information to and *intelligibility* of external stakeholders can simplify and accelerate external life-cycle assessments, registration and approval processes (see Fig. 2), and foster collaborative actions to pursue sustainability objectives (e.g., open science approaches).

In general, *intelligibility* is central to AI-powered R&D, because it constitutes a proethical condition for enabling or imparing judgments of *beneficence*, *non-maleficence*, *justice*, and *autonomy* (Turilli & Floridi, 2009). Understanding the functionalities of AI (i.e., *intelligibility*) can inform evaluations of the other principles by comprehending if and how AI benefits (*beneficence*) or harms (*non-maleficence*) individuals and society in a fair and unbiased way (*justice*) and by drawing conclusions about whether to delegate decisions to AI systems (*autonomy*) (Floridi et al., 2018).

On the other hand, *accountability* focusses on who is responsible for the way AI works, that is, the ethical dimension of *explicability* (Floridi et al., 2018). It is narrowly related to *intelligibility* (e.g.,Coeckelbergh, 2020; Lepri et al., 2018; Martin, 2019; Morley et al., 2020), since judgments about *accountability* necessitate a certain understanding of the underlying processes of AI systems and applications (i.e., *intelligibility*) (Lepri et al., 2018). *Accountability* can create shared responsibility within the organization and responsibility towards external stakeholders, which is particularly relevant in ethically salient contexts. It can be backward-looking, that is, who is ascribed responsibility when something goes wrong, and forward-looking, that is, how can AI systems be designed and used responsibly (Coeckelbergh, 2020). Both views matter for the AI-based R&D of ethically controversial products like pesticides and prompt that human researchers have to be kept in the loop, oversee R&D processes, and anticipate and foresee ethical issues (e.g., adverse effects of substances) for the time being.

Taken together, *explicability* of AI-based R&D is pivotal to meet the global challenge of sustainable development and develop joint actions, and it accounts for both the AI-for-good-perspective (Taddeo & Floridi, 2018) and the collaborative, open-science and transparency stance in ecological and sustainability research (Bausch et al., 2014; Rüegg et al., 2014; Seele, 2016).

## Conclusion

Chemical R&D can be fundamental to solutions that underpin and accelerate sustainable development. Since sustainability initiatives and research always have an ethical dimension (e.g., Schneider et al., 2019; Zagonari, 2020), chemical R&D that are powered by AI and pursue sustainable products and solutions have to be particularly open and explicit about guiding ethical principles and the alignment with existing guidelines (Vinuesa et al., 2020). In light of the rapid advancement of AI, chemical R&D will contribute to the development of sustainable substances and products in the future (e.g., biopesticides; Pretty, 2018) by means of sustainable and resource-efficient R&D processes. Particularly, self-driving laboratories provide promising opportunities (e.g., Häse et al., 2019; Muratov et al., 2020), although human researchers have to remain in the loop for the time being, particularly, in ethically salient research contexts. Notwithstanding, researchers might “soon address challenges that previously were simply considered to be prohibitively complex or demanding, such as automatized experimentation or synthesis of new materials and molecules on demand” (von Lilienthal & Burke, 2020, p. 3). AI can be a game changer to address sustainable development and climate change (Kaplan & Haenlein, 2020), and through chemical R&D, the fuel of AI can be added to the fire of sustainability efforts.

Respective scientific data and knowledge are irreplaceable in a volatile, uncertain, complex, and ambiguous environment, and key conduit to knowledge discovery, integration, and innovation (Rüegg et al., 2014; Wilkinson et al., 2016). Hence, insights generated in the course of AI development and refinement to foster sustainability and related sustainability research findings can be considered a social good. Therefore, a more collaborative, open science approach should be preferred to restrictive proprietary and institutional boundaries on the one hand. On the other hand, scientific data should be managed and potentially made accessible to facilitate seamless re-use and collaboration opportunities to tackle the global challenge of sustainable development and climate change.
